# A Motivational Perspective on Job Insecurity: Relationships Between Job Insecurity, Intrinsic Motivation, and Performance and Behavioral Outcomes

**DOI:** 10.3390/ijerph16101812

**Published:** 2019-05-22

**Authors:** Yuhyung Shin, Won-Moo Hur, Tae Won Moon, Soomi Lee

**Affiliations:** 1 School of Business, Hanyang University, 17 Haengdang-dong, Seongdong-gu, Seoul 133-791, Korea; yuhyung@hanyang.ac.kr (Y.S.); meleesky@naver.com (S.L.); 2 College of Business Administration, Inha University, 100 Inha-ro, Michuhol-gu, Incheon 22212, Korea; 3 College of Business Administration, Hongik University, Seoul 04066, Korea; twmoon@hongik.ac.kr

**Keywords:** job insecurity, intrinsic motivation, job performance, OCB, mediation analysis

## Abstract

As a result of the global economic recession over the past decade, employees have been exposed to constant threats of job insecurity. Despite having conducted extensive research on job insecurity, scholars have paid little attention to the motivational processes underlying employees’ reactions to job insecurity. The purpose of the present study is to examine the relationship between job insecurity, intrinsic motivation, and performance and behavioral outcomes. Drawing on self-determination theory (SDT), we propose a mediated relationship in which job insecurity decreases intrinsic motivation, which, in turn, undermines job performance, organizational citizenship behavior (OCB), and change-oriented OCB. To test our propositions, we collected survey-based data from 152 R&D professionals employed in a South Korean manufacturing company. As predicted, job insecurity was negatively related to intrinsic motivation, which, in turn, had a positive relationship with all three outcomes. Furthermore, job insecurity exerted significant indirect effects on job performance, OCB, and change-oriented OCB through intrinsic motivation. These findings affirm SDT, which posits that motivation, as a key intermediary process, affects employees’ reactions to job stressors.

## 1. Introduction

Over the past few decades, challenges in the business realm have resulted in organizational and structural changes, such as mergers, acquisitions, and downsizings [1,2,3]. These changes, coupled with the recent global economic crisis, have led to increased unemployment and caused employees to feel insecure about their jobs [4,5]. Job insecurity refers to the perceived uncertainty about the continuity of one’s employment [6]. Even if the global economy bounces back, job insecurity is expected to remain a continuing threat to employees whose jobs are being replaced by automation, robots, and artificial intelligence [7]. In line with this trend, there is extensive research on the effects of job insecurity on employee and organizational outcomes. This line of research has revealed that employees’ perceptions of job insecurity have negative ramifications on their job and health-related outcomes [8,9,10]. 

While job insecurity research has provided insights into the relationship between job insecurity and various work outcomes, several gaps remain in the literature. First, prior research has concentrated on employee health and well-being, attitudinal outcomes, and performance and behavioral outcomes as the three major outcomes of job insecurity [7]. Job performance and organizational citizenship behavior (OCB) are well-known performance and behavioral outcomes, respectively. Job performance reflects the extent to which employees fulfill their job requirements, whereas OCB refers to discretionary behaviors displayed by employees that go above and beyond their job descriptions [11]. OCB can be further classified into maintenance and change-oriented OCB [12], depending on whether the aim of the discretionary behavior is to maintain the current level of organizational functioning or to change organizational procedures and policies. Change-oriented OCB is future-oriented behavior with which an employee purports to improve one’s work methods or environment [13,14]. Of the three categories of outcomes, job insecurity’s negative relationships with employee health and well-being and attitudinal outcomes have been well established [7,15,16,17]. However, research has yielded conflicting findings on the link between job insecurity and performance and behavioral outcomes. While some meta-analyses have exhibited a weak, negative association between job insecurity and job performance (e.g., Refs. [8,18]), other studies have indicated a null relationship between the two variables (e.g., Refs. [6,9,19,20]). On the basis of the inconsistent findings on the relationship between job insecurity and OCB, Lam et al. [1] proposed and detected that job insecurity has a curvilinear effect on OCB. These mixed findings call for further investigations. In response to this call, our study aims to examine the relationship between job insecurity and the three types of performance and behavioral outcomes (i.e., job performance, OCB, and change-oriented OCB). 

Second, compared with a large body of research on job insecurity and performance outcomes, relatively scant attention has been paid to the effect of job insecurity on OCB. Moreover, extant studies on job insecurity and OCB have mainly focused on maintenance OCB as an outcome of job insecurity [1,21,22,23,24]. However, considering the increasing importance of proactive, innovative behavior in the workplace [25], we find it surprising that virtually no research has examined the effect of job insecurity on change-oriented OCB. Our study will advance the job insecurity research by expanding the scope of job insecurity’s behavioral outcomes and delving into the relationship between job insecurity and different types of OCB. 

Third, researchers have explored intermediary mechanisms to address how job insecurity affects performance and behavioral outcomes; however, they have generally identified cognitive and affective processes, such as perceived control [26], organizational identification [27], work engagement [3,28], and emotional exhaustion [29], as mediators of the relationship between job insecurity and job performance and OCB. No research has identified intrinsic motivation as a mediator between job insecurity and work outcomes. This is a critical omission because intrinsic motivation encompasses cognitive (i.e., challenge seeking) and affective (i.e., task enjoyment) components [30]. Thus, examining the mediating effect of intrinsic motivation is expected to contribute to a comprehensive understanding of the cognitive and affective processes underlying employees’ reactions to job insecurity.

Although recent self-determination theory (SDT) research has begun to attend to the motivational processes underpinning such a relationship, such studies have proposed the frustration of basic psychological needs as the motivational processes that result from job insecurity (e.g., Refs. [26,31,32,33]). Furthermore, self-determined motivation has rarely been explored in the context of job insecurity [31]. To fill this research gap, we present SDT as an explanatory framework for the relationship between job insecurity and performance and behavioral outcomes, and we propose intrinsic motivation (i.e., the willingness or desire to increase effort because of work enjoyment [30]) as a crucial mediator that transmits the negative effect of job insecurity to job performance, OCB, and change-oriented OCB [34]. As such, our study aims to assess the mediating effect of intrinsic motivation on the relationship between job insecurity and performance and behavioral outcomes.

## 2. Theoretical Background and Hypothesis Development

### 2.1. The Relationship between Job Insecurity and Intrinsic Motivation

The basic premise of SDT is that motivation is an impetus to any human behavior. SDT further categorizes individuals’ motivation into extrinsic and intrinsic motivation [35]. The former refers to the desire to exert effort to acquire outcomes that are external to the work itself (e.g., rewards and recognition), whereas the latter refers to the desire to exert effort because of an interest in, and enjoyment of, the work itself [34,35]. In our study, we argue that intrinsic motivation is more important than extrinsic motivation for two reasons. First, while both types of motivation are positively associated with employees’ task and contextual performance (e.g., Ref. [36]), meta-analytic findings have revealed that intrinsic motivation strongly predicts performance outcomes, regardless of the presence of extrinsic rewards [37]. Second, Ryan and Deci [38] claimed that situational and contextual factors heavily influence intrinsic motivation. We reason that job insecurity, as a situational and contextual factor, affects employees’ intrinsic motivation. Therefore, we identify intrinsic motivation as a crucial linking mechanism between job insecurity and performance and behavioral outcomes.

Prior SDT research has shown that job insecurity thwarts the fulfillment of autonomy, relatedness, and competence needs (e.g., Refs. [26,32,33]). Distinct from those findings, our study proposes that job insecurity is detrimental to intrinsic motivation. When employees experience a high level of job insecurity, they lack control over their jobs and become helpless [26]. Moreover, job-insecure employees perceive their work effort to be meaningless. Sensing a loss of control and lack of meaningfulness hinders employees’ full engagement in their work, thereby leading to decreased intrinsic motivation. Job insecurity research has documented that job insecurity results in perceived powerlessness and a lack of control, thereby diminishing work motivation [28]. Thus, in SDT, job insecurity is a situational and contextual factor that undermines intrinsic motivation.

The negative relationship between job insecurity and intrinsic motivation can also be explained by affective events theory (AET) [39] and conservation of resources (COR) theory [40]. AET postulates that negative workplace events dampen employees’ intrinsic motivation by eliciting negative emotions. On the basis of this theory, we can expect job insecurity to decrease employees’ intrinsic motivation by inculcating negative emotions. On the other hand, COR theory holds that individuals strive to obtain and protect valuable resources. Hobfoll defines resources as “objects, personal characteristics, conditions, or energies that are valued by the individual or that serve as a means for attaining those objects, personal characteristics, conditions, or energies” [40], (p. 516). According to the COR framework, when confronted with work stressors, individuals strive to prevent further loss of resources, thereby reducing their investment of energy and motivation [40]. COR theory applied to job-insecurity contexts reveals that employees experiencing a high level of job insecurity have a decreased commitment to their job and decreased motivation to perform it because they perceive these efforts as a waste of resources [41]. As such, SDT, AET, and COR theory altogether predict a negative association between job insecurity and intrinsic motivation. This line of reasoning leads to the following hypothesis:

**Hypothesis** **1:** Job insecurity is negatively related to intrinsic motivation.

### 2.2. The Relationship between Intrinsic Motivation and Performance and Behavioral Outcomes

The SDT literature has reported a positive association between intrinsic motivation and performance and behavioral outcomes [34,38,42,43,44]. Consistent with these findings, we propose that intrinsic motivation has a positive relationship with job performance. Theorists studying SDT have long recognized that intrinsically motivated people remain highly engaged in work activities [45,46] because those activities interest them and provide spontaneous satisfaction [34,47]. Also, individuals with intrinsic motivation display persistence in the accomplishment of tasks and goals [48,49]; this persistence is a critical precondition for successful job performance. Intrinsically motivated individuals tend to persist in enjoyable, purposeful activities [50]. Those individuals spend a lot of effort on and persevere in the accomplishment of intrinsically rewarding tasks to an even greater extent than they are motivated by extrinsic rewards [51]. Furthermore, intrinsic motivation facilitates psychological engagement in and mobilizes energy for continuous work-related effort, which increases the amount of time they devote to their tasks [49]. As a result, they perform better than those lacking intrinsic motivation. Intrinsic motivation has been found to have a positive impact on students’ academic achievement (e.g., Refs. [52,53]) as well as employees’ job performance (e.g., [48,54]). Meta-analytic findings have also demonstrated a moderate-to-strong relationship between intrinsic motivation and job performance [38]. 

The positive association between intrinsic motivation and OCB has also been well established. Intrinsic motivation is known to be a determinant of OCB (e.g., Refs. [54,55,56,57,58,59]). In a similar vein, SDT research has shown that intrinsic motivation encourages prosocial behavior at work by fulfilling needs for autonomy, competence, and relatedness [60]. Theorists studying SDT maintain that because extra-role behaviors, such as OCB, require individuals to exert extra effort beyond their formal duties, intrinsic motivation is pivotal to the engagement in those behaviors. While job performance is obligatory in organizations and accompanied by extrinsic rewards (e.g., salary and incentives), OCB and change-oriented OCB are not always formally rewarded. Therefore, employees are reluctant to perform OCB if they do not derive true enjoyment and inherent satisfaction from their jobs. Intrinsically motivated individuals tend to engage in OCB because of their interest in and enjoyment of the behavior itself, rather than a desire for rewards and recognition [48]. As intrinsic motivation propels individuals to pursue task significance (i.e., a sense of contributing to the broader community) [48,61,62], employees with intrinsic motivation feel a strong desire to benefit the broader community, and this desire leads to increased OCB. Another explanation underlying the relationship between intrinsic motivation and OCB pertains to positive affect. Intrinsic motivation is known to elicit positive affect [63], which is an emotional condition that fosters OCB [64,65]. When individuals are intrinsically motivated, they feel excited, enthusiastic, and attentive, and therefore engage in discretionary behavior that is beneficial to their organization and colleagues. Using this reasoning, we postulate that there is a positive relationship between intrinsic motivation and OCB.

Parallel to job performance and OCB, we expect that a positive relationship exists between intrinsic motivation and change-oriented OCB. Although there is no reported evidence of the link between intrinsic motivation and change-oriented OCB, prior research has demonstrated the existence of a positive relationship between intrinsic motivation and creativity and risk-taking behavior [30,38,47]. The relationship between intrinsic motivation and change-oriented OCB can be explained by cognitive evaluation theory (CET), a mini-theory of SDT. The central logic of CET is that intrinsic motivation has a growth function that is based on dissatisfaction with current functioning and achievement and seeking optimal challenges (e.g., learning new skills and taking risks) [38,51]. From the standpoint of CET, to challenge the current status quo, intrinsically motivated individuals take risks and seek novel ideas [47], both of which exemplify change-oriented OCB. Another explanation is that the positive affect that arises from intrinsic motivation facilitates cognitive flexibility and expands the scope of attention [66], thereby stimulating employees to challenge current work methods and procedures and discover new ways of doing things at work [49]. Furthermore, curiosity and interest in learning, which are often found in intrinsically motivated people, spur the taking of risks and seeking of novel solutions to organizational problems [47,49], and thus, lead to an increase in change-oriented OCB. Our argumentation leads to the following hypothesis:

**Hypothesis** **2:** Intrinsic motivation is positively related to (a) job performance, (b) OCB, and (c) change-oriented OCB.

### 2.3. The Mediating Effect of Intrinsic Motivation

We propose that intrinsic motivation mediates the negative effect of job insecurity on job performance, OCB, and change-oriented OCB. First, drawing on SDT, AET, and COR theory, job insecurity serves as a work stressor that diminishes employees’ intrinsic motivation. When exposed to threats of job insecurity, employees are likely to experience decreased intrinsic motivation. A low level of intrinsic motivation prevents employees from exerting sustained effort on their tasks, thereby impairing their job performance. A lack of intrinsic motivation caused by job insecurity also stifles employees’ desire to benefit their organization and colleagues, which in turn reduces their OCB. Finally, decreased intrinsic motivation stemming from job insecurity impedes the growth function of intrinsic motivation. As a result, employees are reluctant to challenge the status quo and improve their current work processes. Therefore, we posit the following mediation relationships:

**Hypothesis** **3:** Intrinsic motivation mediates the negative relationship between job insecurity and (a) job performance, (b) OCB, and (c) change-oriented OCB.

## 3. Method

### 3.1. Participants and Procedure 

We contacted R&D professionals from a South Korean manufacturer and invited them to participate in our study. Data collection from a single firm can control for the potential confounding effects of business strategies, performance, control systems, and technological availability of firms [67]. For this reason, prior research has gathered data from a single firm (e.g., Ref. [68]). We targeted R&D professionals from the sponsoring manufacturer because their jobs display a high level of task interdependence and non-routineness, which are conditions for the occurrence of OCB and change-oriented OCB, respectively. The researchers distributed surveys to 230 R&D professionals. They were guaranteed anonymity and confidentiality and were instructed to return the completed questionnaire in a sealed envelope to the researchers. Of the 230 contacted, 152 R&D professionals submitted usable questionnaires (response rate = 66.1%). Eighty-two percent of the 152 respondents were male employees. All respondents held graduate degrees. The age distribution was 20–29 years (*n* = 43, 28.3%), 30–39 years (*n* = 81, 53.3%), 40–49 years (*n* = 27, 17.8%), and over 50 years (*n* = 1, 0.6%). The respondents’ job tenure varied: 1–3 years (*n* = 95, 62.5%), 4–6 years (*n* = 36, 23.7%), 7–10 years (*n* = 27, 7.2%), and over 11 years (*n* = 10, 6.6%). To check the equivalence between the study sample and non-respondents, we requested that the human resource managers of the sponsoring manufacturer compare the demographic characteristics of the study sample with those of all R&D professionals in the manufacturer. They confirmed that there were no significant differences between the two groups in terms of gender, age, job tenure, and education.

Our study was conducted in accordance with the 1964 Declaration of Helsinki and its later amendments or comparable ethical standards. Our data collection procedure was in accordance with the ethical standards of the institutional and national research committees. Prior to survey administration, respondents submitted informed consent and were assured that their responses were anonymous and confidential.

### 3.2. Measures

Because the measurement items used in our study were originally written in English, we administered Brislin’s [69] back-translation procedure. Responses were based on a five-point Likert-type scale (1 = strongly disagree, 5 = strongly agree). Table 1 presents a list of the scale items with standardized loadings.

We used four items from De Witte’s [70] and Schreurs, Van Emmerik, Günter, and Germeys’ scales [41] to measure job insecurity. To assess intrinsic motivation, we used Gagné et al.’s [71] intrinsic motivation scale [60]. Job performance was evaluated with three items from Han, Kim, and Hur’s [72] and Williams and Anderson’s [73] in-role performance scales. We used four items from Ko, Moon, and Hur’s scale [74] to measure OCB. To assess change-oriented OCB, we used Choi’s four-item scale [14].

We controlled for gender, age, job tenure, and positive and negative affectivity in all subsequent analyses because they have potential effects on job insecurity (e.g., Refs. [75,76,77,78]), intrinsic motivation (e.g., Refs. [72,74]), and job performance (e.g., Refs. [74,79,80,81]). Moreover, positive affect correlates with intrinsic motivation, OCB, and change-oriented OCB. We used six items from the positive affect (i.e., active, attentive, and inspired) and negative affect (i.e., afraid, hostile, and nervous) schedule (PANAS) Short Form [82] to measure affectivity.

### 3.3. Data Analysis Strategy 

We tested our hypotheses via structural equation modeling (SEM). The statistical software used in our analyses was M-plus 8.2. We chose SEM over path analysis because the latter does not allow for the simultaneous estimation of all parameters. The measurement items for each variable were averaged prior to being entered in SEM. The model fit in SEM was assessed on the basis of the chi-square (χ^2^/df), comparative fit index (CFI), Tucker–Lewis index (TLI), root-mean-square error of approximation (RMSEA), and standardized root-mean-square residual (SRMR) [83]. Hypothesis 1 was tested by estimating the path between job insecurity and intrinsic motivation. Hypothesis 2 was assessed by estimating the paths between intrinsic motivation and job performance, OCB, and change-oriented OCB. To test Hypothesis 3, we performed a 95% bias-corrected bootstrapping analysis (*n* = 20,000; [84]). 

## 4. Results

### 4.1. Test of Reliability and Validity

Table 2 presents the descriptive statistics and correlations of the study variables. All correlations were in the expected direction. More precisely, job insecurity was negatively related to intrinsic motivation, and intrinsic motivation was positively related to job performance, OCB, and change-oriented OCB. We evaluated the reliability of the measurement items using Cronbach’s alpha (see Table 2). The reliability coefficients for the scales ranged from 0.91 to 0.94, exhibiting sufficient levels of reliability [85]. We also conducted a confirmatory factor analysis (CFA) to test the convergent and discriminant validity of the measurement model. As reported in Table 1, the hypothesized seven-factor model (i.e., job insecurity, intrinsic motivation, job performance, OCB, change-oriented OCB, positive affectivity, and negative affectivity) demonstrated a good model fit in an absolute sense (χ^2^/df = 2.07, *p* < 0.05; CFI = 0.93; TLI = 0.91; RMSEA = 0.08; SRMR = 0.05). The factor and item loadings in the measurement model all exceeded 0.79, with all t-values being greater than 22.21, thereby demonstrating the convergent validity of our measures (see Table 1). All measures displayed a high level of reliability, with composite reliabilities ranging from 0.91 to 0.94 (see Table 2). We further evaluated the discriminant validity of the measures, as recommended by Fornell and Larcker [86]. We found that all average variances extracted (AVEs) were higher than the squared correlation between the target variable and any of the other ones (see Table 2). Taken together, these findings confirm that the reliability and validity of our measures were acceptable.

Because self-reported data were used, we needed to check for potential biases resulting from common-method variance (CMV). We conducted Harman’s one-factor analysis as a statistical remedy [87]. The CFA results showed that the one-factor model (χ^2^/df = 9.53; *p* < 0.05, CFI = 0.38, TLI = 0.32, RMSEA = 0.24, SRMR = 0.18) exhibited a worse fit than our measurement model. We also introduced an additional latent common-method factor (LCMF) on which each item in the baseline model could load, in addition to loading on its respective construct. LCMF accounted for 6.7% of the total variance, which is considerably lower than the median method variance (25%) that is generally observed in research that uses self-reported measures [88]. In summary, these findings suggest that CMV was not a serious threat to our data.

### 4.2. Hypothesis Testing

**Figure 1:**
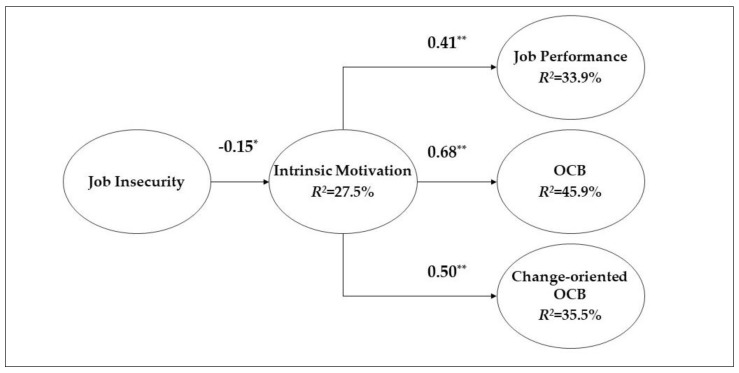
Proposed Research Model and Summary of Results. Note. * *p* < 0.05, ** *p* < 0.01. Path coefficient: unstandardized coefficient. For parsimony, control variables are not included in this figure.

The proposed model demonstrated a good fit with the data (χ^2^/df = 2.02, *p* < 0.05; CFI = 0.92; TLI = 0.90; RMSEA = 0.08; SRMR = 0.07). Figure 1 depicts the variance in intrinsic motivation (27.5%), job performance (33.9%), OCB (45.9%), and change-oriented OCB (35.5%) that was accounted for by their respective predictor.

Hypothesis 1 posits that there is a negative relationship between job insecurity and intrinsic motivation. Figure 1 shows that job insecurity was negatively associated with intrinsic motivation (*b* = −0.15, *p* < 0.05), which supports Hypothesis 1. Hypothesis 2 predicts that positive relationships exist between intrinsic motivation and (a) job performance, (b) OCB, and (c) change-oriented OCB. In support of Hypotheses 2a, 2b, and 2c, we found positive associations between intrinsic motivation and job performance (*b* = 0.41, *p* < 0.01), between intrinsic motivation and OCB (*b* = 0.68, *p* < 0.01), and between intrinsic motivation and change-oriented OCB (*b* = 0.50, *p* < 0.01). 

Hypothesis 3 further postulates that intrinsic motivation has a mediating effect on the relationships between job insecurity and (a) job performance, (b) OCB, and (c) change-oriented OCB. We specified and tested three simple mediation models [89,90,91]. To test this hypothesis, we assessed the significance of the indirect effect of intrinsic motivation using 95% bias-corrected bootstrapping (*N* = 20,000; [84]). We included three additional paths (i.e., job insecurity → job performance, job insecurity → OCB, and job insecurity → change-oriented OCB) [84]. We estimated the proposed mediating effects on the basis of this saturated model. Table 3 reports the estimates of total, direct, and indirect effects for job performance, OCB, and change-oriented OCB. As illustrated in Table 3, while job insecurity had a direct relationship with job performance (*b* = −0.212, *p* < 0.05), it exerted no direct effect on OCB (*b* = −0.076, *p* = n.s.) or change-oriented OCB (*b* = −0.088, *p* = n.s.). As predicted, intrinsic motivation significantly mediated the negative relationship between job insecurity and job performance (*b* = −0.054, 95% CI = [−0.156, −0.005]). Intrinsic motivation partially mediated the negative relationships between job insecurity and OCB (*b* = −0.098, 95% CI = [−0.238, −0.001]) and between job insecurity and change-oriented OCB (*b* = −0.072, 95% CI = [−0.187, −0.005]). These findings lend support to Hypotheses 3a, 3b, and 3c. 

## 5. Discussion

The purpose of this study is to examine the relationship between job insecurity, intrinsic motivation, and performance and behavioral outcomes. More specifically, we propose that intrinsic motivation mediates the relationship between job insecurity and job performance, OCB, and change-oriented OCB. The results of our analyses support all hypotheses. As predicted, we detected a negative relationship between job insecurity and intrinsic motivation and positive relationships between intrinsic motivation and job performance, OCB, and change-oriented OCB. Furthermore, job insecurity exerted a significant, indirect effect on job performance, OCB, and change-oriented OCB through intrinsic motivation. While the proposed mediating effect was supported, the causal relationship between job insecurity, intrinsic motivation, and performance and behavioral outcomes should be interpreted with caution. That is, because the independent variable, mediator, and dependent variables were measured simultaneously, we cannot ascertain the causal direction between the variables. For instance, it is plausible that high performers are intrinsically motivated. Likewise, employees who frequently engage in OCB and change-oriented OCB are likely to be intrinsically motivated. In addition, high performers are less susceptible to threats of job insecurity than low performers, so they perceive a lower level of job insecurity. Accordingly, the causal relationship between job insecurity, intrinsic motivation, and performance and behavioral outcomes warrants further empirical investigations. In the next sections, we discuss the theoretical and practical implications of the present findings.

### 5.1. Theoretical Implications

The present findings make several contributions to the literature on job insecurity. First, our analysis supports the mediating effect of intrinsic motivation and thus suggests that SDT is a relevant framework to explain the effect of job insecurity. COR theory has long prevailed as the theoretical framework to address the relationship between job insecurity and work outcomes. Conversely, several recent studies have adopted an SDT lens to examine the effect of job insecurity (e.g., Refs. [26,31,32,33]. These studies have mainly focused on the frustration of basic psychological needs as the outcome of job insecurity and have rarely explored the role of intrinsic motivation. However, in addition to basic psychological needs, self-determined motivation must be included in this type of research to reach a better understanding of job insecurity from an SDT perspective. Our study corroborates the basic tenets of SDT by demonstrating intrinsic motivation as a key mediator that links job insecurity to performance and behavioral outcomes. By proposing and validating SDT as an overarching framework for delineating the effect of job insecurity on performance and behavioral outcomes, our study complements prior research, which has been dominated by COR theory.

Second, our findings reveal that intrinsic motivation exerted a significant mediating effect on the relationship between job insecurity and performance and behavioral outcomes. Prior research has attended to either cognitive (e.g., perceived control and organizational identification) or affective processes (e.g., emotional exhaustion and work engagement) as mediators of the job insecurity-work outcomes relationship (e.g., Refs. [3,26,27,28,29,92]). Intrinsic motivation encompasses both cognitive and affective components, accounting for why studying the effects of job insecurity through intrinsic motivation can result in a fuller picture of how job insecurity affects employees’ work outcomes. Prior research has ascribed the mixed findings on the job insecurity-job performance relationship to the negligence of mediators and has presented the inclusion of mediators as a potential solution to the inconsistencies (e.g., Refs. [3,26,27,92]). We expand those mediators by treating intrinsic motivation as a crucial mediator of the job insecurity–job performance relationship. That is, when feeling insecure in their jobs, employees do not view their work as interesting and enjoyable, which is the primary reason that their job performance deteriorates. Thus, the job insecurity-job performance relationship cannot be understood completely without considering the underlying motivational processes.

Third, while we found no direct relationship between job insecurity and OCB and change-oriented OCB, we observed a significant, negative association between job insecurity and job performance (see Table 3). These findings indicate that job insecurity is more harmful to in-role performance than to extra-role behavior. As suggested in prior research, job insecurity may not exert an adverse effect on OCB because employees who are experiencing a high degree of job insecurity may take a proactive stance to cope with the crisis. For this reason, Lam et al. [1] noted that OCB can be pronounced in contexts with high job insecurity. That may explain the absence in our data of a direct, negative relationship between job insecurity and OCB and change-oriented OCB. So far, job insecurity research has yielded inconsistent findings on the relationships between job insecurity and performance and behavioral outcomes. Our research helps to resolve such inconsistencies by demonstrating the significant, negative effect of job insecurity on job performance. It also enhances our understanding of the link between job insecurity, performance, and behavioral outcomes by uncovering the differential effect of job insecurity on different performance and behavioral outcomes. 

Although we did not detect a direct link between job insecurity and change-oriented OCB, job insecurity was found to have an indirect effect on change-oriented OCB through intrinsic motivation, which adds a novel insight to the current understanding of job insecurity effects. Change-oriented OCB is distinct from OCB, which maintains the current organizational function and status quo; thus, the former needs to be considered independently of OCB in job insecurity research. Furthermore, in light of the increasing complexity and volatility in the business realm, employees’ proactivity and risk-taking behavior influence organizational effectiveness and innovation [13,80,93,94]. Thus, the inclusion of change-oriented OCB as an outcome of job insecurity is timely and necessary. However, the lack of a direct relationship between job insecurity and change-oriented OCB calls for more research into that relationship. As hinted by Lam et al.’s research [1], job crisis situations may motivate employees to make constructive suggestions and change the current organizational situation. Therefore, the link between job insecurity and change-oriented OCB, as well as its mediating processes, needs to be better determined in future research. 

### 5.2. Practical Implications

In addition to the theoretical implications, our study has managerial implications for practitioners. On the basis of the finding that job insecurity hurts job performance, organizational leaders who are aiming to improve their employees’ job performance need to decrease employees’ job insecurity perceptions. Although job insecurity is inevitable in today’s organizations, there are several ways to diminish the level of job insecurity that employees perceive in the workplace. First, because job insecurity perceptions are heightened in uncertain and unpredictable surroundings [95], managers should reduce uncertainty and increase predictability by clarifying organizational policies, procedures, and performance goals and standards [41]. Second, considering that social support [41,96,97] mitigates the negative impact of job insecurity on job performance, organizations should encourage supportive behavior among employees and cultivate a supportive, cooperative work climate even under threats of job insecurity [98,99]. Our findings highlight the fact that intrinsic motivation transmits the effects of job insecurity to performance and behavioral outcomes. Considering the positive relationship between intrinsic motivation and job performance, OCB, and change-oriented OCB, enhancing intrinsic motivation is an important means to fueling those positive outcomes. Intrinsic motivation can be boosted by assigning jobs to employees that fit their interests, needs, and abilities and by designing challenging, engaging tasks [49]. Organizational leaders should recognize individual employees’ needs and interests when designing their jobs to be more motivating. For example, organizations may want to consider adopting job crafting interventions to better engage employees and help them find meaning in their work [100]. We also recommend that managers exercise empowering leadership to enhance employees’ intrinsic motivation [101]. More specifically, they need to provide employees with autonomy so that they can decide on their work goals, processes, and schedules [37,85], all of which are conducive to self-determined motivation [38]. Finally, it is critical for organizational leaders to provide employees with supportive, developmental feedback to help them feel intrinsically motivated at work.

### 5.3. Limitations 

The present findings should be interpreted in light of the following limitations. First, as noted earlier, the cross-sectional nature of our data hinders the inference of a strong causal relationship between the variables. While the job insecurity literature has generally indicated a causal flow from job insecurity to employee outcomes rather than vice versa [10], there is still insufficient causal evidence of a link between job insecurity and employee outcomes. Thus, we call for more longitudinal research to resolve the causality issue related to job insecurity [102]. 

Second, the use of self-reported measures may limit the empirical rigor of our study. We demonstrated that CMV was not a serious issue in our data; however, self-reported measures of performance and behavioral outcomes are vulnerable to rater biases, such as social desirability and evaluation apprehension. Indeed, our respondents provided favorable ratings of their own job performance and OCB, as indicated by the high means for those variables (*M* = 3.80 for job performance; *M* = 3.77 for OCB). This suggests the possibility that job performance and OCB were overestimated by social desirability and evaluation apprehension. For this reason, researchers have warned against the use of self-reports when assessing performance and behavioral outcomes. While self-reporting can accurately capture individuals’ internal states, such as perceptions and motivation, we recommend other rated measures of job performance, OCB, and change-oriented OCB in future research.

Third, while the present findings expose intrinsic motivation as a linking mechanism between job insecurity and performance and behavioral outcomes, there are other motivational variables that may underlie the job insecurity-outcomes link. In addition to intrinsic motivation, autonomous and controlled motivations are key motivational mechanisms in SDT [38]. Therefore, a potential future research agenda would ideally include the exploration of the mediating roles of autonomous and controlled motivations in job-insecurity contexts. In addition, we note that there are potential moderators that can strengthen or weaken the relationship between job insecurity, intrinsic motivation, and outcome variables. The SDT literature highlights the effects of an interplay between intrinsic and extrinsic motivation on work outcomes [26,27]; this raises intriguing research questions, such as whether extrinsic motivation attenuates the negative impact of job insecurity on intrinsic motivation and whether extrinsic motivation strengthens the positive effect of intrinsic motivation on work outcomes. 

Fourth, we collected data from a single manufacturer to control for potential firm effects, and that method may restrict the generalizability of the study findings. While R&D presents a context in which OCB and change-oriented OCB are likely to occur, the characteristics of R&D professionals (e.g., educational level and task type) may differ from those of other employees. Therefore, one should exercise caution when generalizing the present findings to employees who perform functions other than R&D. It should also be noted that 86% of our sample held job tenure that was less than 7 years. Given that the negative effect of job insecurity is more detrimental to employees with shorter job tenure than those with longer job tenure [75,76,77,78], the relatively short tenure of our sample may have affected the study findings, even though job tenure was controlled for in the analyses. Therefore, we suggest future research that aims to validate our findings in a more tenure-balanced sample. 

Finally, we conducted our study in South Korea, where job insecurity is a serious social phenomenon because of a long-term economic recession; thus, the negative effect of job insecurity on motivation and performance and behavioral outcomes may be stronger in South Korea than elsewhere. Although it is apparent that economic recession and unemployment have been worldwide issues over the past decade, the findings of the current research need to be replicated in different countries to enhance their generalizability. 

## 6. Conclusions

The aim of our work was to explore a motivational mechanism underpinning the relationship between job insecurity and performance and behavioral outcomes. The results of surveys collected from R&D professionals demonstrate that job insecurity has a negative effect on employees’ job performance, OCB, and change-oriented OCB by harming their intrinsic motivation. By revealing intrinsic motivation as a crucial motivational process that links job insecurity to performance and behavioral outcomes, our research offers a motivational perspective to the current job insecurity research. Investigations into different self-determined motivations and boundary conditions, as well as the utilization of a more rigorous methodology, can deepen the knowledge gained from the present study. 

## Figures and Tables

**Table 1:** Factor Analytic Results of Measurement Items.

Construct	Measurement Items	*λ*
Job insecurity	I am sure that I will be able to keep my job. ®	0.88
	There is a risk that I will lose my present job in the near future.	0.90
	I feel uncertain about the future of my job.	0.86
	I think that I will lose my job in the near future.	0.90
Intrinsic motivation	Because I enjoy this work very much.	0.80
	Because I have fun doing my job.	0.93
	For the moments of pleasure that this job brings me.	0.97
Job performance	I adequately completed my assigned duties.	0.88
	I fulfilled the responsibilities specified in my job description.	0.94
	I met the formal performance requirements of my job.	0.86
OCB	I demonstrate behaviors that are consistent with the promise of the organization I work for.	0.89
	I consider the impact on my organization before communicating or taking action in any situation.	0.93
	I show extra initiative to ensure that my behavior remains consistent with the promise of the organization I work for.	0.86
	I am always interested to learn about my organization and what it means for me in my role.	0.87
Change-oriented OCB	I frequently come up with new ideas or new work methods to perform my task.	0.82
	I often suggest work improvement ideas to others.	0.88
	I often suggest changes to unproductive rules or policies.	0.79
	I often change the way I work to improve efficiency.	0.89
Positive Affectivity	Active	0.85
	Attentive	0.92
	Inspired	0.95
Negative Affectivity	Afraid	0.85
	Hostile	0.88
	Nervous	0.93
χ ^2^_(231)_ = 477.74; *p* < 0.05, comparative fit index (CFI) = 0.93, Tucker–Lewis index (TLI) = 0.92, root-mean-square error of approximation (RMSEA) = 0.08, standardized root-mean-square residual (SRMR) = 0.05		

**Notes**. All items measured on a scale ranging from 1 strongly disagree to 5 strongly agree. All factor loadings were significant (*p* < 0.01). ®: reversed code.

**Table 2:** Means, Standard Deviations, and Correlations.

Variables	Mean	SD	α	CR	1	2	3	4	5	6	7
1. Positive Affectivity	2.84	1.01	0.93	0.93	**0.82**						
2. Negative Affectivity	2.48	1.01	0.91	0.92	−0.40 **	**0.79**					
3. Job insecurity	2.37	0.91	0.93	0.93	−0.03	0.34 **	**0.74**				
4. Intrinsic Motivation	3.55	0.89	0.93	0.93	0.43 **	−0.19 *	−0.16 ^†^	**0.82**			
5. Job performance	3.80	0.70	0.92	0.92	0.34 **	0.01	−0.21 *	0.50 **	**0.80**		
6. OCB	3.77	0.71	0.94	0.94	0.27 **	−0.09	−0.16 ^†^	0.63 **	0.67 **	**0.79**	
7. Change-oriented OCB	3.38	0.80	0.91	0.91	0.22 **	0.08	−0.08	0.44 **	0.44 **	0.63 **	**0.72**

***Note***. ^†^*p* < 0.10, * *p* < 0.05, ** *p* < 0.01. Bold numbers along the diagonal are the AVEs (average variances extracted). CR = composite reliability. Categorical variables (i.e., gender, age, job tenure) are omitted. OCB = organizational citizenship behavior.

**Table 3:** Effects for Mediation Model.

Effect	Path	Effect *(b)*	95% CI_low_	95% CI_high_
	Job Insecurity → Job Performance			
Total Effect	Job Insecurity → Job Performance	−0.265	−0.399	−0.145
Indirect Effect	Job Insecurity → Intrinsic Motivation → Job Performance	−0.054	−0.156	−0.005
Direct Effect	Job Insecurity → Job Performance	−0.212	−0.344	−0.067
	Job Insecurity → OCB			
Total Effect	Job Insecurity → OCB	−0.174	−0.331	−0.033
Indirect Effect	Job Insecurity → Intrinsic Motivation → OCB	−0.098	−0.238	−0.001
Direct Effect	Job Insecurity → OCB	−0.076	−0.222	0.067
	Job Insecurity → Change-oriented OCB			
Total Effect	Job Insecurity → Change-oriented OCB	−0.160	−0.345	−0.015
Indirect Effect	Job Insecurity → Intrinsic Motivation → Change-oriented OCB	−0.072	−0.187	−0.005
Direct Effect	Job Insecurity → Change-oriented OCB	−0.088	−0.240	0.035
